# Menkes Kinky Hair Syndrome: A Rare Neurodegenerative Disease

**DOI:** 10.1155/2012/684309

**Published:** 2012-08-07

**Authors:** Rozil Gandhi, Ritu Kakkar, Sajeev Rajan, Rashmi Bhangale, Shrinivas Desai

**Affiliations:** ^1^Department of Radiology, Tata Memorial Hospital, Parel, Mumbai 400012, India; ^2^Department of Radiology, Jaslok Hospital and Research Center, Mumbai 400026, India

## Abstract

Menkes kinky hair disease is a rare X-linked recessive disease nearly exclusively affecting males who present at 2-3 months of age due to abnormal functioning of copper-dependent enzymes due to deficiency of copper. Here, we describe a completely worked-up case of a 4-month-old male infant with very typical history and radiological features confirmed by biochemical and trichoanalysis. The initially seen asymmetric cortical and subcortical T2 hyperintensities in cerebral and cerebellar hemispheres converted into symmetrical diffuse cerebral and predominantly cerebellar atrophy with uniform loss of both white and grey matter on follow-up MRI. Also, subdural hemorrhages of various sizes and different stages and tortuosity of larger proximal intracranial vessels with distal narrowing were identified. Ours is a completely worked-up proven case of Menkes kinky hair disease (MKHD) with history, electroencephalography, biochemical, trichoanalysis, and MRI findings. This is a good teaching case and shows importance of clinical examination and biochemistry as complimentary to MRI. Tortuous intracranial arteries with blocked major vessels are found only in this disease, thus stressing the value of MR Angiography in these patients.

## 1. Case Report

A four-month-old male infant born out of a non-consanguineous marriage at 33 weeks of gestation with perinatal history of cephalhaematoma and hyperbilirubinemia in the neonatal period presented with history of partial seizures and altered sensorium since 4 days which improved with anticonvulsants after 24–48 hours.

Electroencephalogram showed theta-delta range asymmetric background activity with intermittently sharp waves, sharp waves and slow waves seen over left hemisphere, and no further addition by photic stimulation. Metabolic workup done at the time was normal. Initial imaging workup with CT scan of brain revealed mild cerebellar atrophy with area of asymmetrical hypodensities in bilateral posterior parietal subcortical white matter (Figure 2). Subsequent magnetic resonance imaging showed asymmetric T2WI hyperintensities in bilateral temporoparietal cortex and subcortical white matter regions with involvement of the insular cortex with prominence of cerebellar folia suggesting cerebellar atrophy (Figure 3). At this point MRI diagnosis of urea cycle defects like citrullinemia and biotinidase deficiency were considered as differentials. Child was treated with biotin with no clinical improvement.

**Figure 1:**
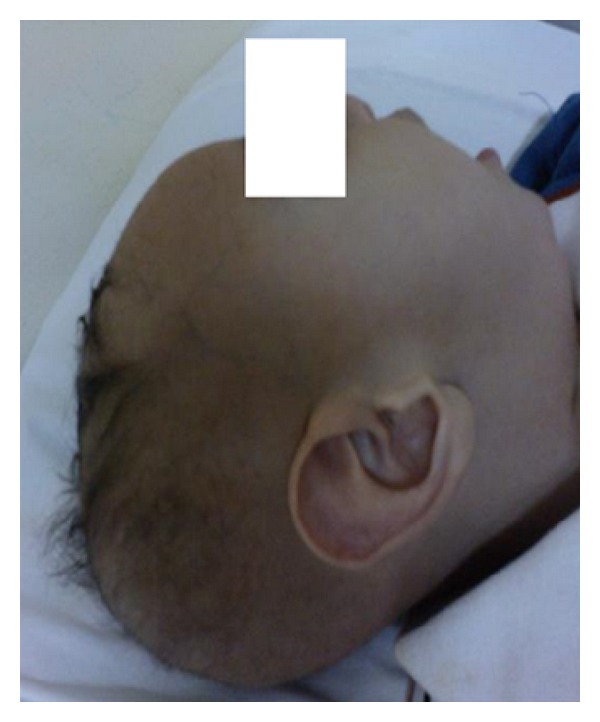
Characteristic phenotypical appearance of baby—fuzzy sparse thin scalp hairs, fair complexion.

**Figure 2:**
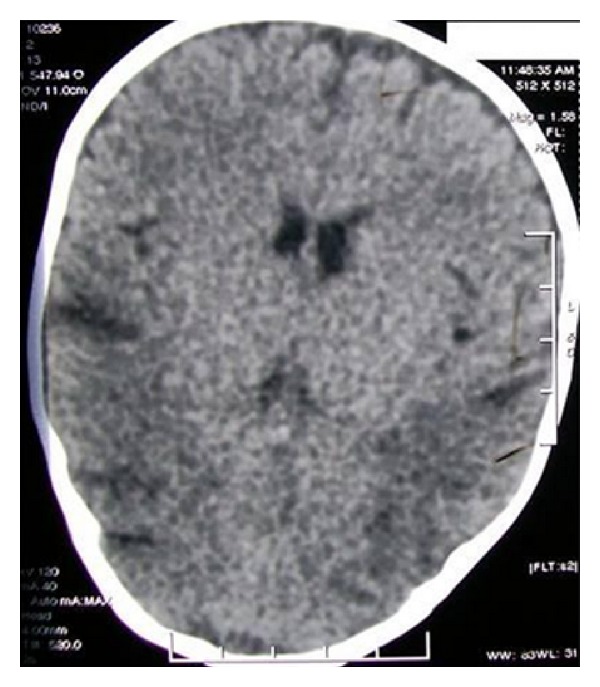
Axial computerized tomography scan shows hypodensities in bilateral posterior parietal subcortical white matter.

Child continued to progress with convulsions and delayed milestones till 8 months of age at which time, on repeat examination, he had reduced tone, inguinal hernia, and poor hair growth (Figure 1).

Metabolic workup revealed increased lactate, decreased serum copper –22 ugm% (N: 75–160), and copper oxidase levels –0.10 OD (N: 0.20–0.55).

**Figure 3:**
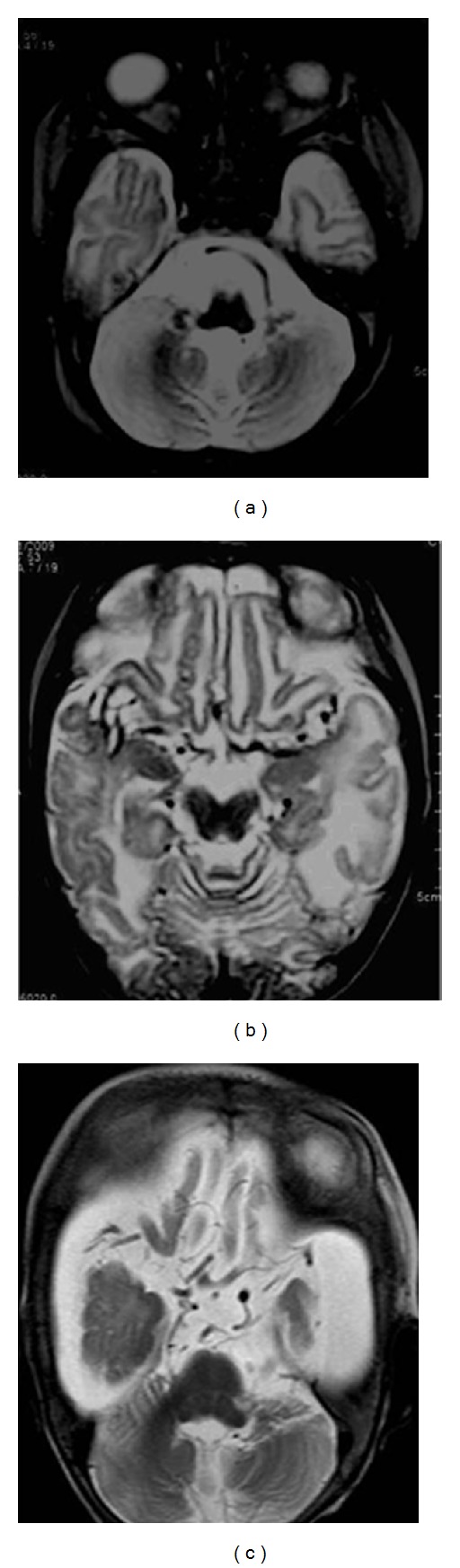
Atrophy of the cerebellum with prominence of cerebellar folia. Asymmetric cortical and subcortical hyperintensities on the axial T2WI predominantly in bilateral temporoparietal regions with involvement of the insular cortex.

**Figure 4:**
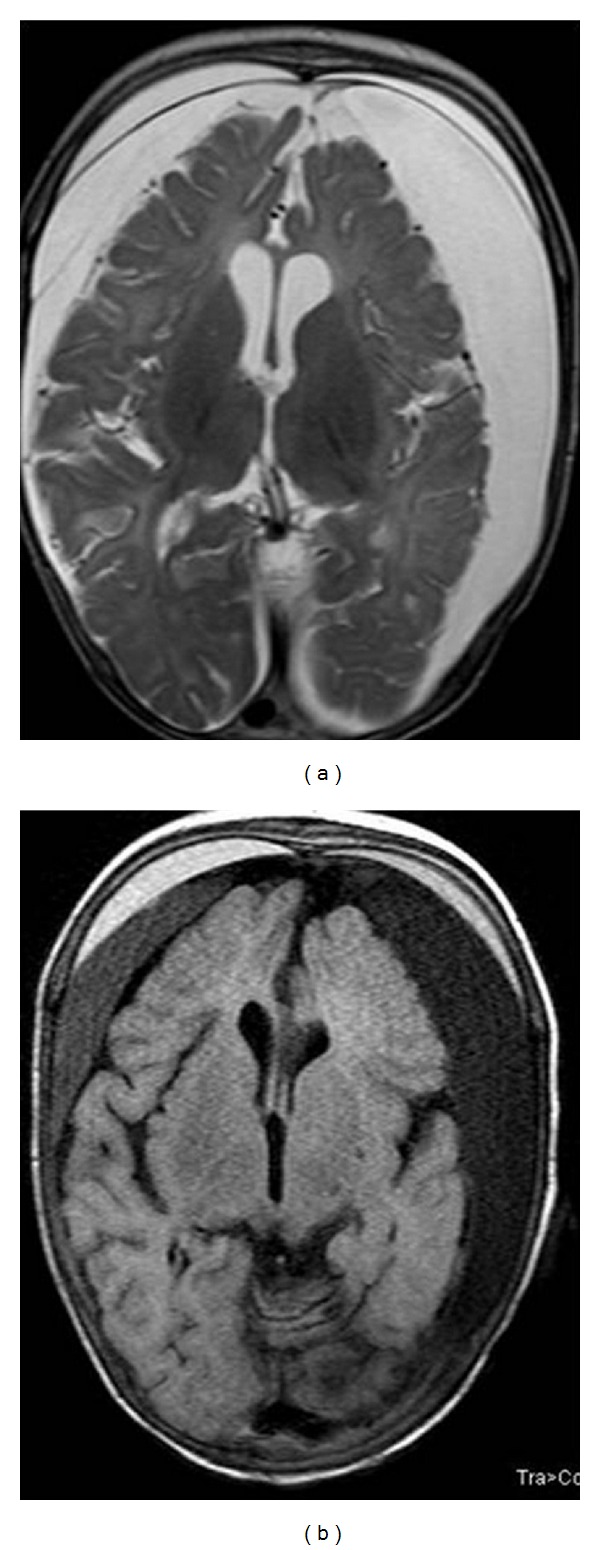
T2(TR: 5000, TE: 102) and fluid-attenuated inversion recovery (TR: 9000, TE: 2500) weighted axial image of brain on Siemens 3 Tesla TIM Trio machine showing bilateral subdural collections of differential intensities suggesting different ages of these hemorrhages.

**Figure 5:**
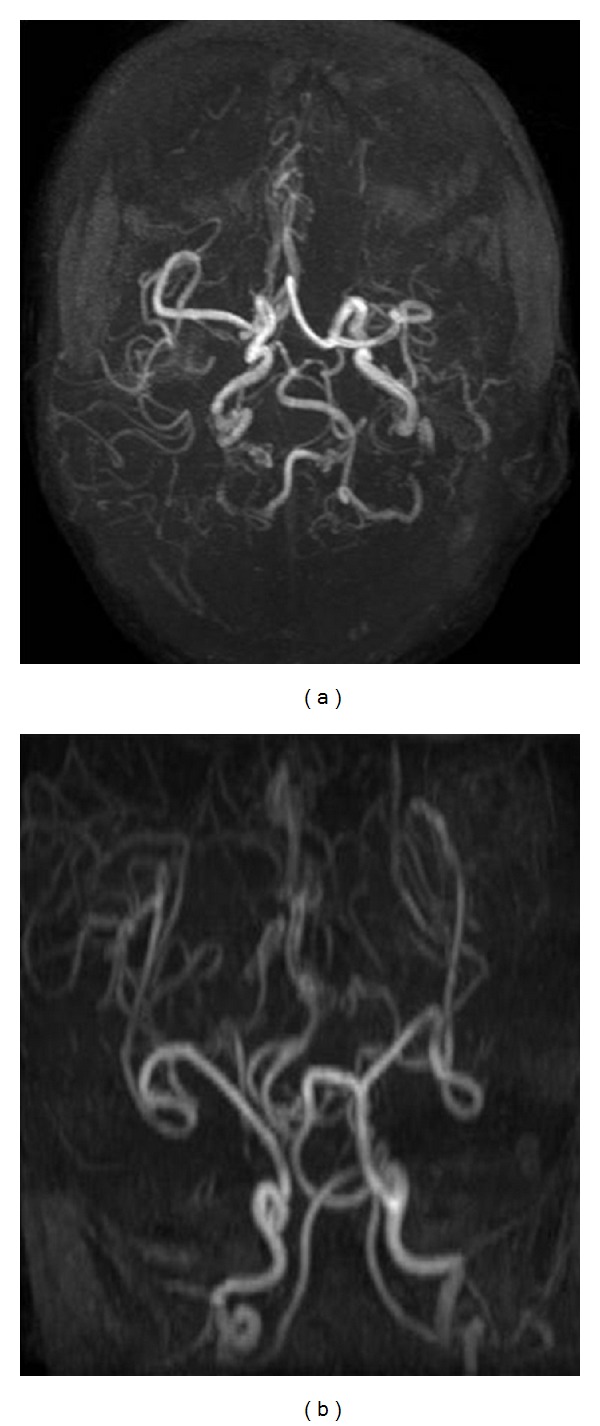
MR angiography: Axial and coronal maximum intensity projection image of brain on Siemens 3 Tesla TIM Trio machine showing tortuous intracranial vessels (TR: 21.4, TE: 3.8).

MRI showed diffuse bilateral white matter hyperintensities on T2-weighted sequences. Symmetrical cerebral and cerebellar atrophy was seen, the latter being more prominent (Figure 3). Extradural and subdural hemorrhages of various stages were identified, one showing fluid-fluid levels in high parietal region (Figure 4). On MR angiography, tortuosity of internal carotid arteries, proximal middle cerebral arteries with distal narrowing were seen (Figure 5).

**Figure 6:**
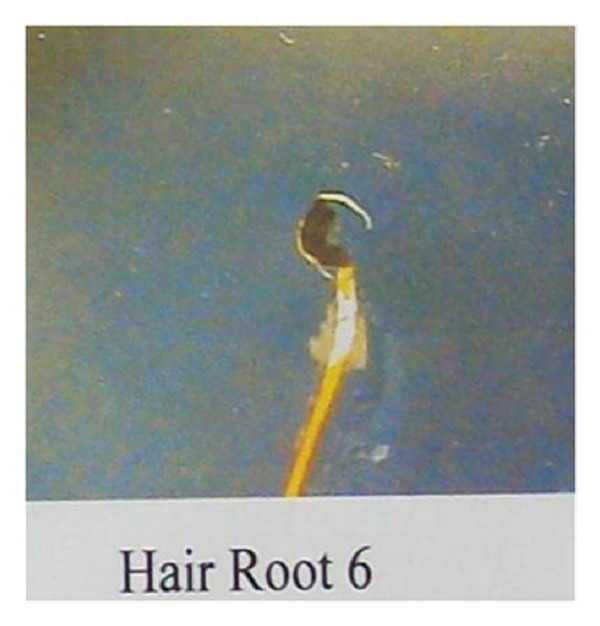
Trichoanalysis showing pili torti.

Trichoanalysis report suggested weak shape of anagen bulb, weak hair shaft with presence of sheath with anagen-telogen ratio of epilated hair: A-6 and T-4, and diagnosis showed pili torti hair which can be present in both Menkes kinky hair syndrome and twisting hair dystrophy (Figure 6).

## 2. Discussion

Menkes kinky hair disease (MKHD) or trichopoliodystrophy is a progressive neurodegenerative disorder with an incidence of 1 case per 300000 [1]. It nearly exclusively affects males presenting at an age of 6–8 weeks [2, 3]. It is an X-linked recessive disease with locus on Xq13.3, who present at 2-3 months of age [1–3]. There is deficiency of multiple copper-dependent enzymes which leads to a primary defect affecting copper transport that begins with impaired absorption at the intestinal level and continues with failed utilization and handling of whatever copper conveyed to other cells in the body [2].

### 2.1. Clinical Features

The patients with Menkes disease are preterm or term delivered babies with nonspecific findings like large cephalhematomas, hypothermia, hypoglycemia, and jaundice in perinatal period. There is development of progressive hypotonia, loss of previously obtained developmental milestones, seizures, myoclonic jerks, failure to thrive, poor weight gain, loose skin, pectus excavatum, urinary bladder diverticula, and appearance of the characteristic short, sparse, coarse wiry hair—pili torti—by the time the individual with Menkes kinky hair disease is aged 4-5 months. Death usually occurs by the time the individual with Menkes kinky hair disease is aged 3 years mostly due to respiratory failure [2]. Early diagnosis is important for genetic counseling although no therapy appears effective [3, 4].

On *radiographs*, long bones show osteoporosis, metaphyseal spurring, periosteal reaction, and scalloping of the posterior aspects of the vertebral bodies [5].

The diagnostic investigation of choice is *MR brain* which shows diffuse symmetrical brain matter atrophy predominantly affecting cerebellum; bilateral diffuse white matter hyperintensities are seen on diffusion, T2, and FLAIR images. Subdural hemorrhages of different stages and sizes are generally seen [6–8].

MRI at four months of age shows intense bilateral hyperintensities of white matter, very similar to diseases primarily affecting white matter. Hypointense areas on T1-weighted and hyperintense areas on T2-weighted images with volume loss of brain matter suggesting atrophy, necrosis, and gliosis of white matter may be identified before the phenotypic alterations. In such cases, the arteriopathy with vascular insufficiency probably leads to a deficient myelination process, mainly in semioval centers and long tracts [9].

One unusual case report showed symmetric deep peri-ventricular white matter lesions with diffuse cortical atrophy. Previously reported lesions were usually asymmetric, and they involved lobar white matter rather than deep white matter. These lesions may demonstrate persistent restricted diffusion which may be due to diffuse white matter ischemia as a result of tortuous intracranial vessels [10]. The angiographic alterations are better visualized after three months of age. Large vessels tortuosities with corkscrew pattern and distal narrowing cause ischemia as well seen on MR angiography [6].

The most important condition in the differential diagnosis is the shaken baby syndrome. The combination of subdural hygromas and hematomas of different ages and bone fractures is very suggestive of this diagnosis. However, the clinical picture with skin and hair abnormalities, tortuosity of the arteries on MRI, and the presence of other bone abnormalities should help in the differentiation. Differential diagnosis also includes Leigh's disease (subacute necrotizing encephalopathy), phenylketonuria, and certain diseases with specific hair findings. The low concentrations of copper and ceruloplasmin are however typical only of Menkes disease [6].

### 2.2. Treatment

Intravenous copper and copper histidine therapy has been tried [4]. There is a case report of a boy with classic MD that was diagnosed at birth attending elementary school in 2002 at 8 years of age. Thus, early diagnosis is very essential [6].

## References

[1] Suguru I *Menkes Kinky Hair Disease*.

[2] Kaler StephenG *Menkes Kinky Hair Disease: Differential Diagnoses & Workup*.

[3] Datta KA, Ghosh T, Nayak K, Ghosh M (2008). Menkes kinky hair disease: a case report. *Cases Journal*.

[4] Lee ES, Ryoo JW, Choi SD, Cho JM, Kwon HS, Shin SH (2007). Diffusion-weighted MR imaging of unusual white matter lesion in a patient with Menkes disease. *Korean Journal of Radiology*.

[5] Kabra M, Gangakhedkar AK, Pasi GR, Manoj R, Verma IC (1996). Menkes’ kinky hair disease: new considerations. *Indian Pediatrics*.

[6] Santos MLG, Teixeira SC, Vilanova CLP (2001). Case report of an uncommon presentation with white matter lesions. Menkes disease. *Arquivos de Neuro-Psiquiatria*.

[7] Fister P, Raku J, Primec RZ, Strazisar GB (2006). Menkes kinky hair disease (Menkes syndrome): a case report. *Acta Dermatovenerologica Alpina, Pannonica et Adriatica*.

[8] Ghofrani M, Pour HHS (1999). Menke’s syndrome. *Medical Journal of Iranian Hospital*.

[9] Jacobs DS, Smith AS, Finelli DA, Lanzieri CF, Wiznitzer M (1993). Menkes kinky hair disease: characteristic MR angiographic findings. *American Journal of Neuroradiology*.

[10] Bindu PS (2007). Menkes kinky hair presenting as myoclonic seizures neuroimaging and EEG observations. *Karnataka Journal of Child Neurology*.

